# Identification of Synovial Lymphatics in the TMJ and their Roles in Arthritis and Pain

**DOI:** 10.21203/rs.3.rs-6683299/v1

**Published:** 2025-05-30

**Authors:** Jian-Fu Chen, Yang Shu, Qing Chang, Ziying Lin, Pao-Fen Ko, Jingyi Chen, Feixiang Chen, Zhen Zhao

**Affiliations:** University of Southern California

**Keywords:** lymphatics, temporomandibular joint, arthritis, pain, mice

## Abstract

Temporomandibular joint (TMJ) arthritis is a craniofacial disorder characterized by joint dysfunction and orofacial pain. Lymphatic regulation and function in the TMJ remain unknown. Using genetic reporter mice, human tissues, tissue clearing, 3D volume imaging, and functional studies, we identified a synovial lymphatic system in the TMJ. In a mouse model of TMJ arthritis, inflammation induces extensive lymphatic remodeling and leads to synovial lymphatic dysfunctions, including decreases in synovial efflux and lymph node fluid drainage. Functional genetics and single-cell RNA sequencing (scRNA-seq) revealed that lymphatic deficiency induces a population of fibrosis-associated macrophages and enhances inflammation, exacerbating cartilage defects, bone loss, synovitis, and pain behaviors in TMJ arthritis mice. Conversely, lymphatic function promotion via a hydrogel-mediated VEGF-C delivery reduced TMJ pain, inflammation, and arthritis-like pathogenesis. For the first time, we identified synovial lymphatics in the TMJ and found that lymphatic dysfunction drives TMJ arthritis and pain, suggesting its potential as a therapeutic target.

## INTRODUCTION

Temporomandibular disorders (TMDs) are a subset of painful craniofacial disorders that involve the TMJ, masticatory muscles, and surrounding tissues^1^. TMDs are estimated to affect approximately 31% of adults and 11% of children^2^. Around 15% of TMD cases continuously progress, show resistance to treatment, and result in chronic pain, making it the second most common musculoskeletal pain condition^3^. TMJ arthritis and TMJ osteoarthritis (TMJOA) represent joint-specific forms of TMDs, characterized by joint inflammation, dysfunction, and degeneration. Both conditions significantly affect quality of life due to arthralgia and restricted mobility^4,5^. Patients with TMJOA usually have a history of arthralgia and a popping noise while performing jaw functions^6-8^. Individuals with arthritis experience severe and usually unremitting pain that can be both physically disabling and emotionally distressing, hampering their potential recovery^9^. While pain is the major reason for patients to pursue medical treatment, the underlying mechanisms and effective management strategies of that pain are still unclear^10,11^.

The lymphatic system is a network of vessels that is present in virtually every organ of the body, complementary to the cardiovascular system. It is composed of blind-ended, highly branched capillaries that function to take up interstitial fluid (ISF), large molecules, and leukocytes from peripheral tissues^12-14^. These capillaries serve as entrance points into lymphatic circulation, playing a crucial role in preventing the accumulation of fluids and immune cells within local tissue^12,13^. Lymph is transported through collecting lymphatic vessels to draining lymph nodes, and is returned into blood circulation via major lymphatic ducts, such as the thoracic duct and the right lymphatic duct^15^. Lymphatic vessels are lined with a single layer of lymphatic endothelial cells (LECs), which express the homeobox transcription factor prospero-related homeobox 1 (Prox1) and receptor tyrosine kinase vascular endothelial growth factor (Vegf) receptor 3 (Vegfr3)^12^. Prox1 is a master regulator of lymphangiogenesis, and its heterozygous mice have been used as a genetic model of lymphatic vessel malfunction^16,17^. The Vegf-c ligand interacts with its receptor Vegfr3 to regulate lymphatic growth and function, which are promoted or inhibited by ectopic expression of Vegf-c or Vegf-c/d trap, respectively^13,18^.

In collecting lymphatic vessels, endothelial cells exhibit an elongated morphology and are connected by continuous junctions known as “zipper junctions.” These vessels are enveloped by a thick basement membrane and layers of contractile lymphatic smooth muscle cells (LSMCs), which limit permeability and support unidirectional lymph flow with the help of intraluminal valves^19^. Capillary lymphatic vessels are composed of LECs and a thin basement membrane, lacking mural cell coverage. These cells are connected by discontinuous, button-like junctions that facilitate high permeability, allowing efficient passage of solutes and immune cells into the lymphatic system^20,21^. Lymphangiogenesis and lymphatic vessel remodeling are frequently observed in inflammation-related biological processes and diseases. For example, the immune-interacting subtype of Ptx3-positive LECs recruit pro-lymphangiogenic macrophages to promote progressive lymphatic overgrowth^22^. The pathophysiological functions of the lymphatic system in inflammatory disorders such as TMJ arthritis remain poorly understood.

Previous studies suggest that impaired lymphatic functions are involved in the pathogenesis of inflammatory knee joint diseases, such as osteoarthritis (OA) and rheumatoid arthritis (RA)^23-25^. Targeting lymphatic vessels to restore drainage, enhance lymphangiogenesis, or resolve inflammation is proposed as a therapeutic strategy for the management of knee joint arthritis^23,26^. However, the TMJ differs from the knee joint in terms of developmental origin, morphology, structural composition, and function^27,28^. It remains unknown if and where lymphatic vessels occur and how they are regulated and function in the TMJ under pathophysiological conditions. In this study, we identified synovial lymphatics in the TMJ. In TMJ arthritis mouse models, lymphatic vessels undergo extensive lymphangiogenesis, coupled with inflammation and drainage dysfunctions. Functional studies revealed that lymphatic dysregulation drives TMJ arthritis and pain, highlighting its potential as a therapeutic target.

## RESULTS

### TMJ lymphatic vessels are identified at synovial tissues.

**Figure 1:**
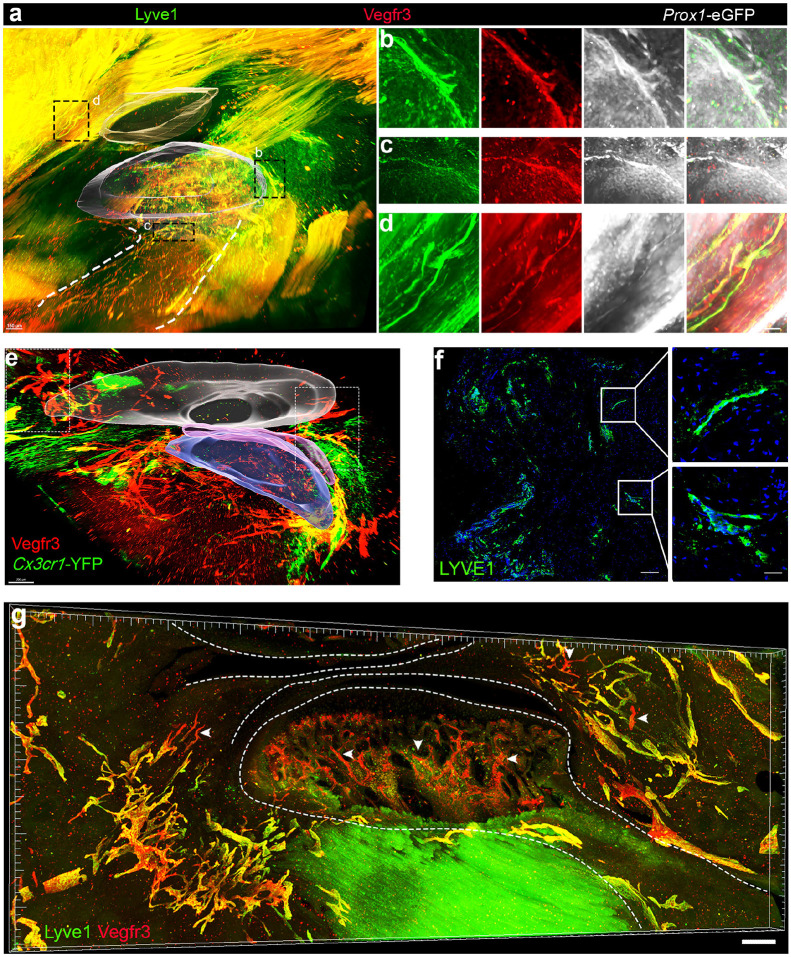
Identification of lymphatic vessels in TMJ. (a) Confocal imaging of cleared TMJ stained with antibodies against Lyve1 (green), Vegfr3 (red), and GFP (white) from adult *Prox1-*EGFP mice. Scale bar: 150 μm. (b-d) High magnification images of the lymphatic vessels in the representative posterior synovial tissue region (b), deep inner region (c), and surrounding muscle (d). Scale bars: 50 μm. (e) Confocal imaging of tissue cleared TMJ stained with Vegfr3 and YFP from adult *Cx3cr1-*YFP mice. Scale bar: 200 μm. (f) Immunofluorescence imaging of human TMJ synovial sections stained with antibodies against LYVE1 (green). DAPI stains nuclei (blue). Scale bars: 100 and 10 μm. (g) Confocal imaging of the 100 μm TMJ section after CUBIC clearing stained with antibodies against Lyve1 and Vegfr3 from WT mice at 3 weeks post-CFA injection. White arrowheads point to Vegfr3+;LYVE1− blood vessels in condyle bone marrow and synovial tissues. Scale bar: 200 pm.

Previous studies reported the distribution and alteration of lymphatic vessels in the knee joints of normal and osteoarthritic mice^29^. Here, we sought to identify and investigate the distribution of lymphatic vessels in the TMJ. Toward this aim, we used *Prox1-*EGFP mice in combination with antibodies against lymphatic vessel markers, including lymphatic vessel endothelial hyaluronan receptor 1 (Lyve1) and Vegfr3. An immunolabeling-enabled iDISCO tissue clearing method was applied to adult mouse TMJs, followed by confocal imaging and 3D reconstruction to generate whole mount images of the TMJ (Fig. 1 a). High magnification images showed that lymphatic vessels co-express Prox1, Lyve1, and Vegfr3, displaying tube-like structures, confirming the lymphatic vessel identities. Moreover, lymphatic vessels were mainly found in the surrounding muscle, anterior, posterior, and superior synovial tissues, as well as the superior retro-discal tissue (RDT) of the TMJ (Fig. 1 b-d). It has been reported that tissue-resident macrophages regulate lymphatic vessel growth and patterning in the developing heart^30^. To investigate the lymphatic vessel-macrophage axis, we used *Cx3cr1-*YFP mice to label resident macrophages and Vegfr3 to mark vascular endothelial cells, followed by iDISCO tissue clearance and confocal imaging of the whole adult mouse TMJ (Fig. 1 e). There are extensive Vegfr3-positive and tube-like vessels at the synovial tissues and surrounding muscles, where Cx3cr1-positive resident macrophages are adjacent to vessel cells (Fig. 1e, Supplementary Video 1).

Whether or not lymphatic vessels occur in the cortical bone or bone marrow is an unsettled issue. Vegfr3 could label blood or lymphatic vessels in a tissue-dependent manner. To achieve a higher resolution imaging and investigate whether Vegfr3-positive vessels in condyle bone are blood or lymphatic vessels, we performed CUBIC (clear, unobstructed brain/body imaging cocktails and computational analysis) tissue clearance of 100 μm-thick TMJ sections from mice exposed to Complete Freund's Adjuvant (CFA), described later as our TMJ arthritis models. Lyve1- and Vegfr3-double positive lymphatic vessels are mainly located in the anterior and posterior synovial tissues (Fig. 1 g, Supplementary Video S2), where Vegfr3+;Lyve1− blood vessels occur less frequent than lymphatic vessels (white arrowheads in synovial tissues). It is clear that Vegfr3-positive vessels in condyle bones are Lyve1 negative (white arrowheads in condyle), suggesting of the absence of lymphatic vessels in condyle bone or bone marrow. Lastly, human TMJ synovial tissues from the arthroscopy surgery of TMJ pain patients confirmed LYVE1− positive tube-like lymphatic vessels (Fig. 1f). Together, Lyve1^+^;Vegfr3^+^ double-positive tube-like structures are identified as lymphatic vessels, which are mainly located at synovial tissues under TMJ healthy and arthritis pathological conditions.

### Lymphangiogenesis and lymphatic vessel remodeling are coupled with inflammation in mouse arthritic TMJ.

**Figure 2:**
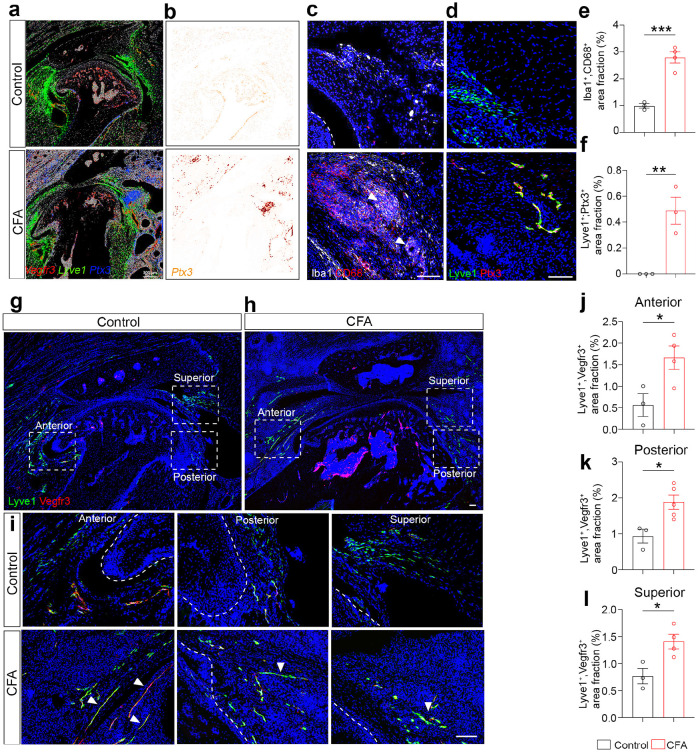
Lymphangiogenesis and lymphatic vessel remodeling are coupled with inflammation in arthritic TMJ. (a,b) seqFISH images of TMJs stained with RNA probes *Vegfr3* (red), *Lyve1* (green), and *Ptx3* (blue or grey). Scale bar: 300 μm. (c, d) Immunofluorescence imaging of the superior regions of TMJ sagittal sections stained with antibodies against Iba1 (white) and CD68 (red), as well as Lyve1 (green) and Ptx3 (red). DAPI stains nuclei (blue). White arrowheads denote Iba1 and CD68 double-positive regions. Scale bars: 50 μm. (e, f) Quantification of the area fraction of Iba1^+^ and CD68^+^ double-positive macrophages or Lyve1^+^ and Ptx3^+^ double-positive inflammatory lymphatic vessels in the superior area. (g-i) Confocal imaging of TMJ sagittal sections stained with antibodies against Lyve1 (green) and Vegfr3 (red). DAPI stains nuclei (blue). Scale bars: 100 μm. Images in i are enlargements of dashed box regions of TMJ in g-h at the anterior, posterior, and superior regions. White arrowheads denote elongated and tube-like lymphatic vessels. Scale bars: 50 μm. (j-l) Quantification of the area fraction of Lyve1^+^ and Vegfr3^+^ lymphatic vessels in the different TMJ areas. All data are represented as mean ± SEM calculated by Student’s *t*-test, n ≥ 3 mice, **p<0.01, ***p<0.001.

To investigate lymphatic vessels under TMJ pathological conditions, we turned our attention to animal models of TMDs. Complete Freund's Adjuvant (CFA) is an inflammatory agent that causes joint inflammation, a pathological trigger for painful TMJOA^31^. We utilized our previously established mouse models of CFA-induced inflammatory TMJOA, which recapitulate osteoarthritic pathologies and orofacial pain behavior in patients^3^. CFA intra-articular injection was performed to induce TMJ arthritis. IHC staining confirmed the joint inflammation, as evidenced by increased macrophage activation marker CD68 and pan-macrophage marker Iba1 in arthritic TMJ (Fig. 2c, e). We conducted spatial transcriptomic analysis of control and CFA-treated TMJs at single-cell levels using Sequential Fluorescence In Situ Hybridization (seqFISH)^32^, as described in a separate study. Using our seqFISH data, we confirmed the synovial tissue-enriched location of lymphatic vessels labeled by *Vegfr3* and *Lyve1* double-positive RNAscope signals (Fig. 2a). Furthermore, *Vegfr3* and *Lyve1* double-positive signals were increased in the CFA compared to the control TMJs (Fig. 2a), suggesting increased lymphangiogenesis. To confirm the lymphangiogenesis phenotype, we performed side-by-side IHC staining using antibodies against Vegfr3 and Lyve1 on control and CFA TMJ sections (Fig. 2g, h). There was a substantial increase of lymphatic vessels at the anterior, posterior, and superior synovial regions of the TMJ in CFA-treated TMJs compared to controls (Fig. 2i-l). In addition to increased immune-reacting areas, CFA-treated TMJ synovial tissues house more and longer tube-like vessels stained by Vegfr3 and Lyve1 (Fig. 2i-l), which is consistent with our CUBIC cleared 100 μm-thick TMJ sections (Fig. 1 g and Supplementary Video S2). Ptx3 was used to mark a subpopulation of inflammatory LECs, characterized by their ability of recruiting pro-lymphangiogenic macrophages to promote lymphatic overgrowth^22,33^. Therefore, we examined our seqFISH data for *Ptx3* and found its upregulation in conjunction with *Vegfr3* and *Lyve1* in the synovial tissue regions in CFA-treated TMJs (Fig. 2b). Next, IHC staining confirmed Ptx3 upregulation in tube-like lymphatic vessels in synovial tissues of arthritic TMJ, whereas no Ptx3 signals were detected in control synovial tissues (Fig. 2d, f). Together, these studies revealed inflammatory lymphangiogenesis and lymphatic vessel remodeling in mouse models of TMJ arthritis.

### Lymphatic functions are impaired in mouse arthritic TMJ.

**Figure 3:**
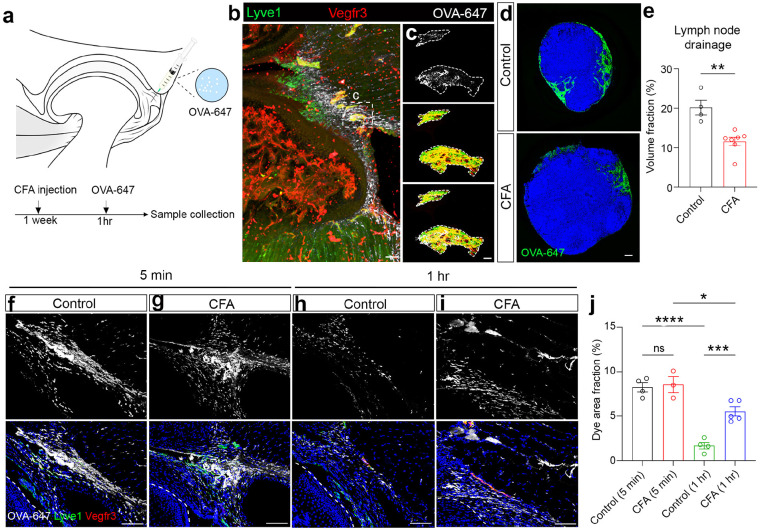
Lymphatic functions are impaired in mouse arthritic TMJ. (a) Diagram of TMJ lymphatic drainage experiment. (b, c) Confocal imaging of the sagittal section (Z-stack of 100 μm) of adult mouse TMJ stained with antibodies against Lyve1 (green) and Vegfr3 (red) after intra-articular injection of OVA-647 (white). DAPI stains nuclei (blue). Scale bars: 100 and 50 μm. High-magnification images in c represent the lymphatic absorption of OVA-647 proteins. (d) Immunofluorescence imaging of sections of the accessory mandibular lymph nodes with the drainage of OVA-647. Scale bar: 100 μm. (e) Quantification of the volume fraction of OVA-647 dyes in the lymph nodes. (f-i) Immunofluorescence imaging of sagittal TMJ sections stained with antibodies against Lyve1 (green) and Vegfr3 (red) after OVA-647 (white) dye injection into the superior region of TMJ. DAPI stains nuclei (blue). Scale bars: 50 μm. (j) Quantification of the area fraction of OVA-647 dyes in the superior region of TMJ. All data are represented as mean ± SEM calculated by Student’s *t*-test or one-way ANOVA with Tukey post hoc tests, n ≥ 3 mice, *p<0.05, **p<0.01, ***p<0.001, ****p<0.0001.

Inflammation-associated lymphangiogenesis is a double-edged sword and is actively involved in the pathophysiology of various inflammatory disorders^34^. To investigate how lymphatic vessel remodeling is translated into lymphatic function, we examined lymphatic drainage functions of TMJ synovial tissues. About one week following CFA administration, 10 uL of Alexa Fluor 647-conjugated ovalbumin (OVA-647) protein was injected into the superior area of the TMJ, followed by 1 hr of waiting before sample collection (Fig. 3a), which allows dye drainage into the accessory mandibular lymph nodes. To confirm that OVA-647 dye can be absorbed by lymphatic vessels, we performed IHC staining on 100 μm-thick TMJ sections using the CUBIC tissue clearing method. Confocal imaging showed the co-localization of OVA-647 dye with lymphatic vessels labeled by Vegfr3 and Lyve1 in the synovial tissues of the TMJ (Fig. 3b). Next, we evaluated how the pathological condition affects lymphatic drainage capacity. Whole accessory mandibular lymph nodes were harvested and subjected to CUBIC tissue clearing. Confocal imaging and reconstruction results showed that CFA-treated mice exhibited reduced dye drainage to the mandibular lymph nodes compared to control mice (Fig. 3d, e).

The impaired lymphatic drainage function in CFA mice could be due to the disruption of fluid influx (entering synovial capillary lymphatic vessels), fluid efflux (flowing out of capillary synovial lymphatic vessels), or fluid clearance (collecting vessel-mediated flow out from synovial tissues and into lymph nodes). To provide more insights into impaired lymphatic drainage in CFA-treated TMJ, we performed IHC staining of TMJ sections at 5 minutes and 1 hr after dye injection into synovial tissues. There was no significant difference in OVA-647 dye intensity at lymphatic vessel-enriched regions between control and CFA groups at 5 minutes post-injection (Fig. 3f, g, j), suggesting normal synovial fluid influx. In contrast, there was a significant increase of dye retention in the CFA group compared to controls at 1 hr post-injection (Fig. 3h, i, j), suggesting impaired fluid efflux. Together, these studies suggest that inflammation-associated lymphangiogenesis is coupled with impaired fluid efflux, leading to defective lymphatic drainage functions in TMJ arthritis mouse models.

### Lymphatic deficiency exacerbates synovial inflammation and pain behavior in TMJ arthritis mice.

**Figure 4:**
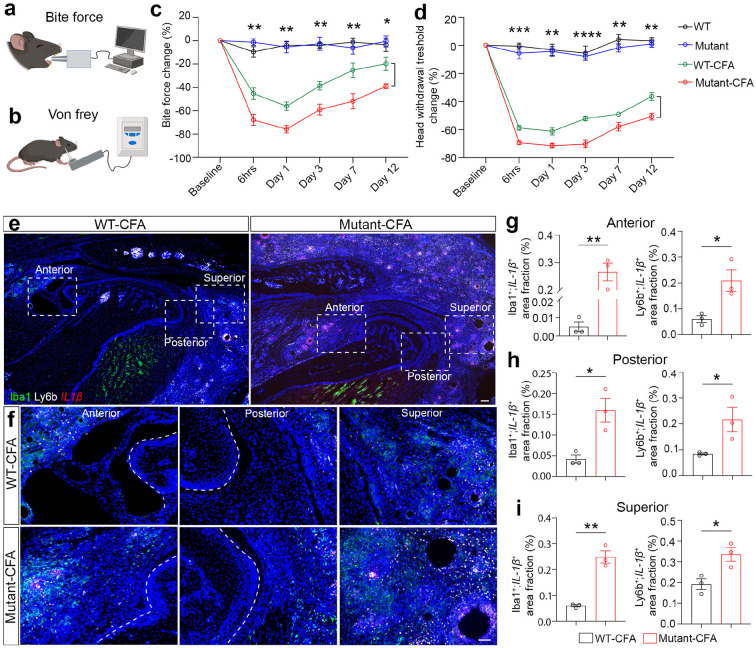
Lymphatic deficiency exacerbates synovial inflammation and pain behavior in TMJ arthritis mice. (a, b) Diagram of bite force and von Frey filament measurement. (c) Quantification of relative bite force values. n = 6 mice (WT), n = 7 mice (*Prox1^+/−^* Mutant), n = 12 (WT-CFA), n = 8 (*Prox1^+/−^* Mutant-CFA). (d) Quantification of head withdrawal threshold measurement. n = 6 mice (WT), n = 7 mice (*Prox1^+/−^* Mutant), n = 12 (WT-CFA), n = 8 (*Prox1*^*+/−*^ Mutant-CFA). (e, f) Combined RNAscope of *IL-1/β* (red) and immunofluorescence staining of Ly6b (white) and Iba1 (green) in different regions surrounding TMJ. DAPI stains nuclei (blue). Scale bars: 100 μm. Images in f are enlargements of dashed box regions in e. Scale bars: 50 μm. (g-i) Quantification of the area fraction of *IL-1β^+^* and Iba1^+^ macrophages as well as *IL-1β^+^* and Ly6b^+^ neutrophils at anterior, posterior, and superior regions of TMJ. All data are represented as mean ± SEM calculated by Student’s *t*-test, n ≥ 3 mice, *p<0.05 and **p<0.01.

CFA-induced TMJ arthritis mice exhibited orofacial pain, synovitis, cartilage remodeling, and bone loss^3^, which recapitulates TMJOA patient phenotypes. To investigate the biological significance of lymphatic remodeling and drainage disruption in TMJ arthritis, we performed functional perturbation of the lymphatic system using *Prox1^+/−^* heterozygous mice. Prox1 is a key transcriptional factor regulating lymphangiogenesis and *Prox1^+/−^* mice have been used as a genetic tool of lymphatic vessel malfunctions^16,17^. Bite force and Von Frey filament assays were used to measure the nociceptive pain behavior on mouse models of TMDs^35^ (Fig. 4a, b). There was no difference in bite force or head withdrawal thresholds between wild-type (WT) and *Prox1^+/−^* mutant mice (Fig. 4c, d), suggesting that lymphatic vessel malfunction in *Prox1^+/−^* mutant mice *per se* did not cause orofacial pain behavior. Under CFA-induced inflammatory conditions, longitudinal quantification showed that mutant mice had more significant reduction in bite force compared to control mice over several days after CFA injection (Fig. 4a, c), suggesting that lymphatic deficiency deteriorates TMJOA pain. Consistent with the bite force assay, Von Frey filament longitudinal quantification showed that mutant mice had a more significant reduction in head withdrawal threshold compared to control mice under joint arthritis conditions (Fig. 4b, d).

To investigate mechanisms underlying pain behavior, we examined inflammation of the TMJ using anterior, posterior, and superior regions as representative areas. We performed RNAscope analysis of inflammatory cytokine *IL-1/β,* in conjunction with IHC staining using antibodies against Iba1 to label macrophages and Ly6b to label neutrophils, respectively. There were more *IL-1β*^+^Iba1 ^+^ double-positive inflammatory macrophages in mutant TMJs compared to WT controls under the same CFA treatment (Fig. 4e-i). Similarly, *IL-1β*^+^Ly6b^+^ neutrophils were increased in mutant TMJs compared to WT controls after CFA treatment (Fig. 4e-i). Consistent with the immune regulation function of the lymphatic system, these results suggest that lymphatic deficiency causes more inflammation and exacerbates orofacial pain in TMJ arthritis mouse models.

### scRNA-Seq analysis reveals fibro-macrophage cell expansion in lymphatic-deficient TMJ arthritis.

To investigate cellular and molecular mechanisms underlying the severe inflammation of lymphatic-deficient TMJs, we performed scRNA-seq analysis to systematically examine major cell types in the TMJ. The TMJ samples with post intra-articular inoculation of CFA were taken from 2-month-old female WT or *Prox1^+/−^* mutant mice (Fig. 5a). Using Seurat 3 R-Package, we obtained ~ 7000–8000 cells with a median of ~ 3000–4000 genes per cell. After unbiased clustering of gene profiles, cell types were identified based on the top differentially expressed genes (DEGs) and known cell-type marker genes. These analyses resulted in 13 primary cell types (Supplementary Fig. 1), including macrophages (*C1qa, C1qb, C1qc*), endothelial cells (*Flt1, Kdr, Pecam1*), mesenchymal cells (*Dcn, Col3a1, Igfbp6*), erythrocytes (*Hba-a1, Car2, Slc4a1*), smooth muscle cells (MSC, *Myh11, Acta2, Tpm2*), neutrophils (*S100a8, Retnlg, Mmp8*), Schwann cells (*Plp1, Mbp, Mpz*), B cells (*Cd79a, Vpreb3, Ighm*), T cells (*Icos, Skap1, Itk*), adipocytes (*Adipoq, Car3, Cfd*), muscle cells (*Pax7, Dmd, Lama2*), chondrocytes (*Col2a1, Acan, Col1a1*), and osteocytes (*Bglap, Runx2).* These cell clusters were relatively well-separated (Fig. 5b), indicative of the high integrity of our scRNA-seq data.

Compared to the WT control, mutant TMJ tends to have an increased proportion of macrophages (Fig. 5b, c), which is consistent with enhanced inflammation in mutant synovial tissues (Fig. 4e-i). To have a more in-depth analysis, we re-clustered the macrophage population and identified four subclusters, including monocytes (*Ccr2, Ly6c2, Ctsg*), M1-like inflammatory macrophages (*Nos2/iNOS, Mmp12, Spp1*), M2-like anti-inflammatory and tissue-repairing macrophages (*Mrc1/CD206, Retnlg, Ccl8*), as well as a new population of fibrosis-associated macrophages (FAMs, Fibro-M) or matrix-producing macrophages (MPMs) (Fig. 5d, g). These FAMs account for most of the increased macrophage population in mutant compared to control TMJs (Fig. 5e) and exhibit a gene program of pro-fibrosis status, extracellular matrix (ECM) generation, and tissue remodeling, as evidenced by the upregulation of *Col1a1, Col1a2, Sparc,* and *Acta2* (Fig. 5f, g). This fibro-M cluster expresses typical macrophage marker *C1qa,* is inflammatory (*Il-1b*), and has an increased expression of ECM gene *Col1a1* (Fig. 5h). Fibrosis, characterized by excessive deposition of ECM components such as collagen, leads to tissue stiffening and impaired joint function, ultimately resulting in ankylosis, one of the end-stage phenotypes of TMJOA^36^. To confirm the pro-fibrosis status of the increased macrophage cluster, we performed Masson’s Trichrome Staining to characterize collagen deposition and reorganization. Under the same CFA-induced joint arthritis condition, mutant synovial tissues contained high levels of cross-linked collagen along with tissue reorganization when compared to controls (Fig. 5i-k). Our scRNA-seq analysis revealed the expansion of macrophages including a new fibrosis- and ECM remodeling-related macrophage subpopulation, which might contribute to synovial tissue inflammation, stiffness, and lymphatic vessel remodeling in the mutant TMJ.

### Lymphatic deficiency exacerbates TMJ arthritis defects in mice.

**Figure 6:**
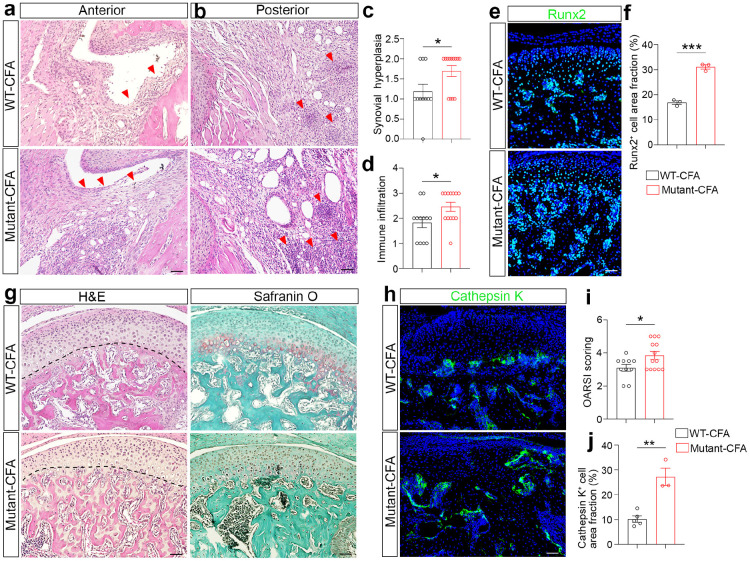
Lymphatic deficiency exacerbates TMJ arthritis defects. (a, b) H&E staining of sagittal TMJ sections after the intra-articular injection of CFA into WT and *Prox1^+/−^* mutant mice. Red arrows in image a denote hyperplastic epithelial lining in the anterior area of TMJ. Red arrowheads in image b denote immune cell infiltration in the posterior area of TMJ. Scale bars: 50 μm. (c, d) Quantification of TMJ synovitis evaluated by Synovitis Scoring System with two assessment criteria, including synovial hyperplasia (c) and immune cell infiltration (d). (e, h) Immunofluorescence imaging of sagittal TMJ sections stained with antibodies against Runx2 or Cathepsin K. DAPI stains nuclei (blue). Scale bars: 50 μm. (f, j) Quantification of the area fraction of Runx2^+^ cells or Cathepsin K^+^ cells in the TMJ condyle. (g) H&E staining and Safranin-O staining of sagittal TMJ sections. Scale bars: 50 μm. (i) Quantification of Osteoarthritis Research Society International score in TMJs. All data are represented as mean ± SEM calculated by Student’s *t*-test, n ≥ 3 mice, *p<0.05, **p<0.01, ***p<0.001.

Increased pain and inflammation prompted us to examine TMJ integrity in *Prox1^+/−^* lymphatic-deficient mice. To this end, we focused on previously characterized CFA-induced inflammatory TMJOA phenotypes, including cartilage remodeling, bone loss, synovitis, and increased OA score^3^. H&E staining was performed on joint paraffin sections at 2 weeks after CFA injection into WT control or *Prox1^+/−^* mutant TMJs. As shown in TMJ anterior regions, mutant mice had more severe synovial membrane hyperplasia (marked by red arrows) compared to control mice (Fig. 6a, c). Focusing on the posterior region, we found that mutant mice exhibited dense inflammatory infiltrates, with some areas forming large follicle-like aggregates (Fig. 5b). In contrast, control TMJs displayed less immune cell infiltration, as well as fewer and smaller follicle-like structures in the synovial tissues (Fig. 5b, d).

To examine the cartilage integrity, we performed H&E staining and found a relatively thinner and disorganized layer of fibrocartilage in mutant mice compared to controls (Fig. 6g). Consistently, Safranin-O staining showed more severe cartilage degeneration-like defects in mutant than control TMJs (Fig. 6g). We employed the Osteoarthritis Research Society International (OARSI) scoring system and found an increased OARSI score in mutant TMJs (Fig. 6i). These results suggest that lymphatic deficiency exacerbates the cartilage defects in arthritic TMJs. Next, we examined osteoblast and osteoclast phenotypes in the TMJ. Phenotypically unstable osteoarthritic chondrocytes pathologically express osteoblast genes such as *Runx2,* which indicates the aberrant cartilage remodeling^37^. There is a significant increase of Runx2-positive cells across the fibrocartilage and bone marrow regions in mutant compared to control TMJs (Fig. 6e, f). In addition, Cathepsin K-positive osteoclasts were robustly increased in mutant bone marrow compared to controls (Fig. 6h, j), suggesting the increased osteoclast activity as the potential underlying mechanism of bone loss in lymphatic-deficient TMJs. Together, these studies suggest that lymphatic deficiency leads to more severe synovitis and TMJ degeneration under arthritic conditions, establishing the causative relationship between lymphatic dysfunction and TMJ arthritis.

### Lymphatic promotion mitigates immune activation and orofacial pain in TMJ arthritis mouse models.

**Figure 7:**
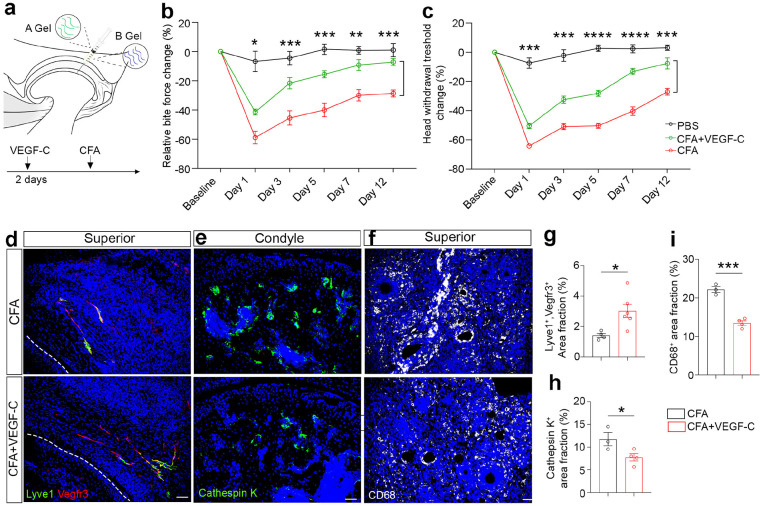
Lymphatic promotion mitigates immune activation and orofacial pain in TMJ arthritis mouse models. (a) Diagram of VEGF-C treatment experiment. (b) Quantification of relative bite force values at different time points. n = 10 mice (PBS), n = 10 mice (CFA), n = 14 (CFA + Hydrogel + VEGF-C). (c) Quantification of head withdrawal threshold measurement at different time points. n = 10 mice (PBS), n = 10 mice (CFA), n = 14 (CFA + Hydrogel + VEGF-C). (d-f) Immunofluorescence staining of TMJ sagittal sections using antibodies against Lyve1 (green) and Vegfr3 (red), Cathepsin K (green), or CD68 (white). DAPI stains nuclei (blue). Scale bars: 50 μm. (g-i) Quantification of the area fraction of Lyve1^+^ and Vegfr3^+^ lymphatic vessels, Cathepsin K^+^ osteoclasts, or CD68^+^ macrophages. All data are represented as mean ± SEM calculated by Student’s *t*-test, n ≥ 3 mice, *p<0.05, ***p<0.001.

The VEGF-C ligand activates its LEC-enriched receptor VEGFR3 to enhance lymphatic growth and functions^12,38^. To promote lymphatic functions, we used the VEGF-C156S recombinant protein (refer to VEGF-C) with a Cys156 to Ser mutation, which yields a lymphangiogenesis-specific form of VEGF-C that specifically binds to VEGFR3^39^. We utilized a degradable, injectable, and sustainable hydrogel as the delivery vehicle of the VEGF-C protein (refer to *DishGef)* (Fig. 7a). This hydrogel is composed of A Gel and B Gel, both of which are liquid that can be mixed with VEGF-C protein thoroughly, followed by the intra-articular injection into the TMJ. Our *in vitro* experiments revealed that A gel and B gel solidify at ~5 minutes after their combination, allowing for the sustained TMJ retention and VEGF-C release to occur at a slow pace. CFA injection causes joint inflammation and swelling, which prevents us from doing a second injection of *DishGel.* Therefore, we injected VEGF-C-containing *DishGel*first, followed by intra-articular CFA injection 2 days later, which is technically feasible (Fig. 7a). IHC staining confirmed the lymphatic promotion after VEGF-C *DishGel* treatment, as evidenced by the increased Lyve1 and Vegfr3 double-positive and tube-like lymphatic vessels (Fig. 7d, g).

Longitudinal measurement showed that VEGF-C treatment significantly enhanced bite force in CFA-treated mice (Fig. 7b), suggesting of pain mitigation and improved TMJ functions. Similarly, the head withdrawal threshold was enhanced after the VEGF-C-containing *DishGel* treatment (Fig. 7c). These results suggest that VEGF-C-mediated lymphatic promotion mitigated pain behavior in TMJ arthritis mice. To examine how lymphatic promotion affects immune cells, we performed IHC staining of TMJ sections using antibodies against Cathepsin K to label osteoclasts and CD68 to label activated macrophages, respectively. VEGF-C treatment had a significant reduction of osteoclasts compared to controls (Fig. 7e, h). Similarly, CD68-labeled macrophages were reduced in VEGF-C-treated mice compared to controls (Fig. 7f, i). Together, these results suggest that lymphatic promotion by VEGF-C-containing *DishGel* decreased immune activation and led to pain mitigation in TMJ arthritic mice.

### Lymphatic promotion ameliorates joint degeneration in TMJ arthritis mouse models.

**Figure 8:**
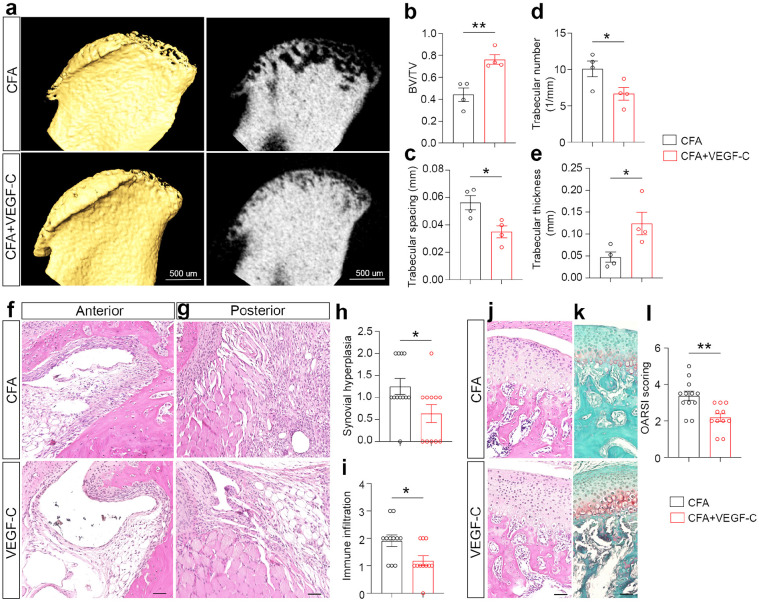
Lymphatic promotion ameliorates joint degeneration in TMJ arthritis mouse models. (a) Micro-CT imaging of the mandibular condyle. Scale bars: 500 μm. (b-e) Quantification analysis of microarchitecture parameters of the subchondral bone. BV/TV: bone volume/total volume; 1/mm: 1 trabecular number per mm region. (f, g) H&E staining of TMJ sagittal sections. Note that VEGF-C-treated mice presented less synovitis, including less hyperplastic epithelial lining and less immune cell infiltration. Scale bars: 50 μm. (h, i) Quantification of TMJ synovitis evaluated by the Synovitis Scoring System with two assessment criteria: synovial hyperplasia (h) and immune cell infiltration (i). (j, k) H&E staining and Safranin-O staining of TMJ sagittal sections. Scale bars: 50 μm. (l) Quantification of Osteoarthritis Research Society International score in TMJs. All data are represented as mean ± SEM calculated by Student’s *t*-test, n ≥ 3 mice, *p<0.05, **p<0.01.

To examine whether lymphatic promotion by VEGF-C could reduce degenerative processes in our mouse model, micro-CT imaging was used to analyze bone architecture at 2 weeks after CFA intra-articular injection. There was a clear morphological change in the subchondral bone in VEGF-C groups compared to controls (Fig. 8a). VEGF-C-treated TMJs exhibited a significant increase in bone volume (bone volume over total volume, BV/TV) and in trabecular thickness (Fig. 8b, e), as well as a robust decrease in trabecular space and trabecular number (Fig. 8c, d). Therefore, VEGF-C-treated TMJ mitigated bone loss in CFA-induced TMJ arthritis mice, possibly due to decreased bone-absorbing osteoclasts as revealed in our previous analyses (Fig. 7e, h). Together, these results suggest that the prevention of subchondral bone loss in arthritic TMJs after lymphatic activation is likely due to decreased osteoclasts.

Next, we performed histopathology analyses to characterize synovitis and cartilage integrity. As exampled in the anterior region, VEGF-C-treated mice had less synovial membrane hyperplasia compared to controls (Fig. 8f, h). We focused on the posterior region and found that VEGF-C-treated mice exhibited less immune cell infiltration compared to controls (Fig. 8g, i). H&E staining and Safranin-O staining revealed that VEGF-C-treated TMJs have an improved cartilage integrity compared to controls (Fig. 8j, k), which is consistent with the OARSI score reduction after VEGF-C-mediated lymphatic promotion in TMJ arthritic mice (Fig. 8l). Together, these findings suggest that lymphatic promotion mitigates synovitis and joint degeneration in TMJ arthritic mice.

## DISCUSSION

Here we identified a synovial lymphatic system in the TMJ. Using TMJ arthritis mouse models, we found that lymphatic vessels undergo extensive lymphangiogenesis and remodeling coupled with lymphatic drainage function disruption during the progression of joint arthritis with pain. Lymphatic dysfunction exacerbates TMJ arthritis and pain likely due to induced interstitial fluid retention and fibro-macrophage activation, which collectively contribute to worsen inflammation, leading to nociceptive activation, pain sensation, and joint degeneration.

We identified lymphatic vessels, mapped their anatomical locations, and characterized their structure and function in TMJs under pathophysiological conditions. We utilized *Prox1-EGFP* genetic mice, coupled with immunolabeling with Lyve1 and Vegfr3 to mark lymphatic vessels, followed by tissue clearance, 3D volume imaging, and imaging reconstruction in control and arthritic TMJ mouse models. Lymphatic vessels are primarily localized at TMJ synovial tissues, where they are adjacent to Cx3cr1-labeled resident macrophages. Consistently, arthritic TMJ displayed robust lymphangiogenesis and lymphatic vessel remodeling, resulting in elongated and tube-like vessels, which is likely attributed to macrophage expansion and their secretion of VEGF-C. In turn, VEGF-C can activate VEGFR3 in LECs and lead to an increase in lymphatic vessel growth. These findings are consistent with previous reports describing lymphatic remodeling and increased lymphangiogenesis under inflammatory conditions^14,34^. Despite their growth, these lymphatic vessels in arthritic TMJs are compromised and dysfunctional, as evidenced by the aberrant expression of inflammatory LEC marker Ptx3, impaired synovial interstitial efflux, and damaged lymph node drainage functions. Future studies should examine lymphatic permeability, contractility, and the transition between lymphatic button and zipper junctions in arthritic TMJs.

Our functional studies showed that the TMJ lymphatic network plays critical roles in regulating fluid homeostasis and immune response. This observation is consistent with findings in knee joints, where lymphatic vessels function to drain cavity space fluid, interstitial fluid, macromolecules, and immune cells, thus preventing inflammation and joint degeneration^29,40,41^. Beyond joint degeneration, our studies found that lymphatic dysfunction exacerbates pain behavior, which is barely examined in previous lymphatic studies of joint. In the *Prox1^+/−^* lymphatic malfunction mice, lymphatic deficiency leads to increased expression of *IL-1β*^+^Iba1 ^+^ macrophages and *IL-1β*^+^Ly6b^+^ neutrophils around the TMJ, which might yield more pro-inflammatory mediators or immune cell infiltration^42,43^. In addition, *Prox1^+/−^* mice displayed more severe arthritic pathological features, including synovitis, subchondral bone remodeling, cartilage degeneration, and fibrosis than control mice under the same CFA treatment. In our lymphatic gain-of-function studies, hydrogel-mediated delivery of VEGF-C to the TMJ alleviated pain behaviors and reduced immune cell activation. Micro-CT and histological analyses further revealed less subchondral bone loss, decreased synovitis, and improved cartilage integrity. Together, these studies established the causative relationship between lymphatic dysfunction and TMJ degeneration with orofacial pain in our TMJ arthritis mouse models. It is important to investigate how lymphatic vessel remodeling and dysfunction in inflammatory TMDs occur in other types of TMJ disorders, such as mechanical and injury models of TMDs.

Our studies identified impaired interstitial fluid flow and induced fibro-macrophage activation as the key underlying mechanisms for TMJ degeneration and pain TMJ arthritis mouse models. Our findings suggest that in response to inflammation, lymphatic vessels remodel and grow to enhance the drainage functions for removing inflammatory mediators from the interstitial tissues. After reaching a certain threshold in the transition from physiological to pathological, lymphatic vessels are impaired in both structure (Ptx3 inflammation) and function (interstitial efflux and lymph node drainage). As a result, inflammatory mediators cannot be effectively removed and are trapped in the interstitial synovial tissues, which in turn impair lymphatic vessels while also activating nociceptors. Our study on lymphatics in the TMJ provides experimental evidence and validates the previous hypothesis of impaired lymphatic drainage and interstitial inflammatory stasis in pain^44^. Not only are inflammatory mediators ineffectively removed, but their generation actually increases due to the rise in fibrosis-associated macrophages. Our scRNA-seq identified a cluster of fibro-macrophages, which are highly enriched with ECM proteins (Col1a1, Col1a2) and pro-inflammatory cytokines (*IL-1β*), and are the most increased macrophage subset after lymphatic dysfunctions. These stromal-interacting macrophages may be protective early on but progress towards fibrosis and stiffness if chronically activated as suggested by their gene expression profile. Using Masson’s Trichrome Staining, we observed severe collagen deposition and fibrotic tissue accumulation in lymphatic-deficient synovial tissues. Our finding is consistent with previous reports of fibrosis as a critical feature in joint degeneration, leading to stiffness, pain, and impaired function^45,46^. The excessive and abnormal deposition of ECM, characterized by increased dense and cross-linked collagen fibrils, disrupts the normal biomechanical environment necessary for joint homeostasis, leading to loss of tissue elasticity and joint mobility^36,47^.

Our studies provide therapeutic implications for targeting lymphatics to mitigate inflammatory TMJ degeneration and pain. Our hydrogel-based *DishGel* delivery of VEGF-C-activated lymphatic growth and mitigated arthritis pathologies and orofacial pain, which provides a feasible and reliable method of functional perturbation of the lymphatic system in the TMJ. It should be noted that our VEGF-C treatment occurred prior to CFA lesion, therefore, lymphatic promotion happened before the onset of TMJ arthritis. We demonstrated that promoting healthy lymphangiogenesis is beneficial in TMJs. The decision of whether the lymphatic system should be activated or inhibited for treatment of TMDs depends on its pathophysiological stage. For example, it has been reported that lymphangiogenesis mediates renal inflammation and fibrosis^48^, which suggests the detrimental effects of pathological lymphangiogenesis. Future studies should investigate the effects of lymphatic activation or inhibition on specific stages and types of TMDs after the phenotype manifestation.

## MATERIALS AND METHODS

### Mouse models.

The C57BL/6J (JAX#000664) mice were obtained from Jackson Laboratory. *Prox1-*EGFP and *Prox1*^*+/−*^ were from Dr. Young-Kwon Hong laboratory. Animals were housed with regulated humidity, temperature, and a 12-hour alternating light-dark environment. Animals were euthanized by carbon dioxide inhalation, with cervical dislocation performed immediately afterward to confirm death. Pain behavioral studies used female mice due to inherent differences between sexes in nociceptive sensitivity, with higher TMJ pain incidence and levels in females. All studies were performed with the approval of the Institutional Animal Care and Use Committee of the University of Southern California.

### CFA intra-articular injection.

The TMJ injection area was identified by palpating the zygomatic arch. A depression located approximately 2 mm anterior to the external auditory canal, beneath the posterior part of the zygomatic arch, served as the anatomical landmark. The needle was inserted until its tip contacted the bone, roughly 2 mm below the surface. Subsequently, 10 μL of CFA (Chondrex, Inc; concentration 5 mg/mL) or fluorescently labeled Ovalbumin (OVA-647; Thermo Fisher; concentration 0.5 mg/mL) were slowly injected into each TMJ capsule. Following injection, the needle was held in place for at least 5 seconds before careful withdrawal. The control group mice underwent bilateral injections using sterile PBS (10 μL per capsule).

### Nociceptive behavior assessment.

Before behavioral testing, mice were habituated in the behavior room for at least 1 hour. Baseline measurements of bite force and head withdrawal thresholds were recorded during a 3-day pre-training phase, prior to intra-articular injections of PBS or CFA. After training, mice typically initiate biting behavior within 10 seconds after exposure to the bite force sensor. Mice that failed to bite spontaneously within 10 seconds after 3 days of training were excluded from the experiment. To measure bite force, each mouse was gently restrained in a modified 50 mL plastic tube, allowing free and comfortable head movement of testing mice. A bite force sensor (YFM-1-100, measurement range 0-100 N) was connected to the NBIT RSD-V2.6.3 software via an NST2000 data acquisition system (Nanjing Shen-yuan-sheng Intelligent Technology Co.) for measuring bite force. The voluntary bite force was recorded over a period of 2 minutes per session, and the five strongest bite amplitudes were averaged to determine the final bite force value.

Head withdrawal thresholds were measured using an electronic von Frey analgesiometer. Mice were habituated for at least 1 hour in wire mesh cages placed within the behavior room prior to testing. The von Frey filament was applied perpendicularly to the TMJ region, and the head withdrawal threshold was defined as the minimum force required to elicit a head withdrawal response. Measurements were conducted for at least five individual trials per animal, separated by 10-second intervals, during approximately 2 minutes of restrained positioning. Threshold values from each trial were averaged to get the final head withdrawal threshold. The detailed bite force and Von Frey assays for TMJ pain were described in our recent video publication^35^.

### Micro-CT (μCT) analysis.

Micro-CT live imaging was performed on a Scanco Medical *μ*CT 50 scanner (Scanco Medical, Switzerland) at the University of Southern California Molecular Imaging Center (90 kVp, 78 μA, 10 μm pixel size). AVIZO 9.4.0 (Thermo Fisher Scientific) was used to perform 3D reconstruction of the TMJ. The microarchitecture parameters of the subchondral bone, including bone volume over total volume (BV/TV), trabecular spacing (TbSp), trabecular thickness (TbTh), and trabecular number (TbN) were analyzed using VGStudio Max3.3 (Volume Graphics, Inc., USA). For each sample, three spherical regions of interest (ROI) with a radius of 0.1 mm were selected at the midpoints of the anterior, middle, and posterior condyle for all measurements. Each dot in the graph quantification represents one sample (n). Student’s t-test was used for statistical analysis. A significance level was set at a *p*-value of 0.05.

### Histology analysis.

The TMJs from mice were dissected, fixed, and processed through decalcification and washing steps. Subsequently, tissues were dehydrated through a graded ethanol series, cleared with xylene, and embedded in paraffin for sectioning. Histological sagittal sections of the TMJs in 5 μm were used to detect histological changes in joints. The severity of the cartilage degeneration of TMJ samples was assessed using Hematoxylin and Eosin (H&E) and Safranin-O staining, according to a modified Osteoarthritis Research Society International (OARSI) scoring system^3,49^. Scores ranged from 0 to 6 points, with 0 representing intact and continuous cartilage surface, evenly distributed matrix, properly oriented chondrocytes with clear boundaries, and no signs of proliferation or cell death. A higher OARSI score is calculated by the degree of unclear borders between cartilage and subchondral bone, uneven cartilage surfaces, and decreased hypertrophic layer thickness. Synovitis was quantified based on the severity of synovial lining hyperplasia and immune cell infiltration. Synovial lining hyperplasia was graded on a scale from 0 to 2. Synovial membrane staining was graded as follows: 0 for 1–3 layers, 1 for 4–6 layers, and 2 for more than 7 layers. Inflammation was graded based on the severity of inflammatory cell infiltration: 0 for no infiltrates, 1 for a few perivascular lymphocytes or plasma cells, 2 for numerous lymphocytes or plasma cells occasionally forming follicle-like aggregates, and 3 for a dense band-like infiltrate or multiple large follicle-like aggregates. Collagen deposits were evaluated using Masson’s Trichrome Staining (Trichrome Stain Kit - Masson's, Statlab, #204183) on 5 μm paraffin sections. Cytoplasm, muscle fibers, and keratin were stained in shades of pink to red, while collagen fibers appeared blue.

### Immunostaining.

TMJ and lymph node sections were prepared for immunofluorescence staining following standard protocols. Lymph nodes were harvested from the neck region and fixed in 4% PFA at 4°C overnight. The samples were then dehydrated in 30% sucrose at 4°C overnight, followed by a 1:1 mixture of 60% sucrose and OCT (Tissue-Tek, Sakura) at 4°C overnight. Tissues were then embedded in OCT on dry ice and sectioned at a thickness of 8 μm. For TMJ cryosections, samples were decalcified in 14% EDTA for at least 10 days, followed by gradual dehydration in 30% sucrose overnight and in a 1:1 mixture of 60% sucrose and OCT at 4°C overnight. The samples were then embedded in OCT, frozen on dry ice, and sectioned at a thickness of 14 μm using a cryostat (Leica CM1850).

Antigen unmasking solution (Vector, H-3300) was used for antigen retrieval. The primary antibodies included Lyve1 (Invitrogen, 14-0443-82), Vegfr3 (R&D, AF743), Cathepsin K (CTSK; 11239-1-AP), Iba1 (FujiFilm, 019-19741), CD68 (Bio-Rad, MCA1957GA), Runx2 (Cell Signaling, 12556), Ptx3 (Invitrogen, PA5-36156), and Ly6b (Bio-Rad, MCA771GT). And Alexa Fluor 488/568/647 (Invitrogen) were used as secondary antibodies. DAPI (Invitrogen; Cat# 62248) was used for nuclear staining. The percentage of positive immunofluorescence signals and area fractions were analyzed using Image J software/Adobe Photoshop. Quantification was performed using three to five sections per mouse. Each dot in the graph represents the mean value of an individual mouse within the group. At least three mice were used for each group or genotype.

### RNAscope staining.

Sample preparation and RNAscope staining were conducted following the standard ACD protocol using the RNAscope^®^ Multiplex Fluorescent Reagent Kit v2 (Cat. No. 323100). Mm-Il1β (316891-C1) probe was used in this study. A negative control probe (Cat. No. 320871) was used for staining and imaging to minimize background signal. RNAscope staining was quantified using ImageJ at 20x magnification and analyzed according to ACD scoring guidelines. At least three mice from independent litters were used for this study.

### CUBIC clearing.

Animals were administered an intra-articular injection of OVA-647. One hour later, samples were collected following the steps below. Following anesthesia, the animals underwent transcardiac perfusion with cold PBS, followed by 4% PFA. The dissected TMJs were further fixed with 4% PFA at 4°C overnight. After thorough rinsing with cold PBS, the samples were decalcified in 20% EDTA (pH 7.4) for 7 days. Subsequently, the decalcified TMJs were dehydrated in 30% sucrose, followed by incubation in a 1:1 mixture of 60% sucrose and OCT. TMJ tissues were then rapidly frozen in Tissue-Plus O.C.T. compound, followed by sectioning at a thickness of 100 μm using a cryostat (Leica, Cat. #CM1950). Slides were processed using the CUBIC tissue clearing method. Samples were first washed three times with PBST, followed by incubation in Tissue-Clearing Reagent CUBIC-L (TCI, T3740) at 37°C for 2 days. After three additional PBST washes, tissues were incubated with Lyve1 and Vegfr3 primary antibodies at 4°C for 48 hours. The samples were then washed three times with PBST at room temperature before being incubated with secondary antibodies at 4°C for 24 hours. Finally, the tissues were immersed in Tissue-Clearing Reagent CUBIC-R (TCI, T3741) overnight at 37°C until they became transparent. Slides were mounted using CUBIC-R solution for imaging.

Lymph node clearing was performed using the same CUBIC protocol as described above. Briefly, animals received OVA-647 injections and were sacrificed at 5 minutes or 1 hour post-injection for tissue collection. Accessory mandibular lymph nodes were isolated and fixed in 4% PFA overnight. Following washes with PBS, the samples were incubated in CUBIC-L solution at 37°C for a minimum of 3 days. After PBS washing, the attached membrane was carefully removed under the microscope. Lymph node nuclei were stained with PI at a 1:1000 dilution, resulting in a pink-to-red coloration after 48 hours. The samples were then washed with PBS before incubation in CUBIC-R for 2 days. Finally, lymph nodes were mounted in CUBIC-R solution on a confocal imaging plate.

### iDISCO tissue clearing.

TMJ samples were post-fixed with 4% PFA/PBS at 4°C overnight and washed twice with PBS for 1 hour each. After rinsing with PBS, the samples were decalcified in 20% EDTA for 7 days. Then, samples were gradually dehydrated through two sequential incubations in 30% ethanol (prepared in ddH_2_O) for 1 hour each followed, by two changes of 50%, 80%, and 100% ethanol, with each step lasting 1 hour. Samples were then treated overnight at 4°C with 5% H_2_O_2_ (Sigma, H1009), followed by progressive rehydration through a graded ethanol-to-water series. Next, samples were incubated in a cold permeabilization solution composed of 25% (w/w) urea, 15% (w/w) glycerol, 15% (w/w) Triton X-100, and 45% (w/v) double-distilled water at 4°C for 5 hours. This was followed by enzyme-mediated digestion using 0.2% (w/v) collagenase (Merck, 10,103,578,001) in PBS at 37°C for 30 minutes under continuous shaking. Samples were then washed twice for 5 minutes each using a solution containing 2% (v/v) FBS (Sigma-Aldrich, F7524) in PBS on a rocking shaker. For the staining, samples were transferred to a fresh blocking solution composed of 10% (v/v) donkey serum (Abcam, ab7475), 10% (v/v) DMSO (Sigma-Aldrich, D5879), and 0.5% (v/v) Triton X-100 in PBS and incubated at 37°C for 20 minutes. After the blocking, tissues were treated with VEGFR3 and GFP primary antibodies (Rockland, 48776) prepared in an antibody dilution buffer containing 2% (v/v) donkey serum, 10% (v/v) DMSO, and 0.5% (v/v) Triton X-100 in PBS. The samples were incubated overnight at 37°C with gentle agitation at 120 rpm, PBS wash, and 2nd antibody treatments. Lastly, tissues were dehydrated using a gradient of ethanol concentrations (30%, 50%, and 80%) for 30 minutes each, transferred to pure methanol for 1 hour, and washed twice with ethyl cinnamate (ECi) (Sigma-Aldrich, 112372) for 5 minutes per wash. Subsequently, tissues were incubated in a clearing solution composed of 80% (v/v) ECi and 20% (v/v) polyethylene glycol (PEG) (Sigma, 447943) under gentle rotation at room temperature for 30–60 minutes before imaging.

### Confocal imaging and imaging process.

Cleared TMJ and lymph node samples were imaged using a Leica STELLARIS 5 confocal microscope. Imaging was performed with a 10x/NA0.3 objective lens (2 mm working distance) and a 63x/NA1.40 objective lens (200 μm working distance). LAS X Life Science Microscope Software (version 1.4.6) was used for image acquisition. The microscope was equipped with fixed laser wavelengths at 405 nm, 488 nm, 561 nm, and 633 nm. Scans were conducted at a zoom factor of 0.75x, utilizing either the 10x/NA0.3 or 63x/NA1.40 objective lens, with a step size of 1 μm under continuous scanning in the 488 nm, 561 nm, and 633 nm channels. To improve visual representation, gamma correction was applied to raw data from the confocal microscope. ImageJ (NIH, http://imagej.nih.gov/ij) was used for file format conversion, while Bitplane Imaris (http://www.bitplane.com/imaris/imaris, version 10.1.1) facilitated 3D reconstructions, manual annotations, quantification, and video generation.

### Library preparation and Single-Cell RNA Sequencing (scRNA-Seq).

TMJ tissues were carefully dissected from WT or *Prox1*^*+/*^ mice after CFA treatment, finely minced in a tissue suspension medium consisting of Minimum Essential Medium (MEM) with 2% FBS, placed in a fresh centrifuge tube containing 10 mL of digestion medium (Collagenase P, 1 mg/mL; Dispase II, 2 mg/mL), and incubated at 37°C under continuous rotation for 25 minutes. Following digestion, tissues were resuspended in suspension medium and centrifuged at 500 g for 10 minutes at 4°C, after which the supernatant was discarded. The pellet was then resuspended in 4 mL of DNase I solution (2 U/mL in MEM) and incubated at 37°C for 10 minutes. To facilitate tissue dissociation, suspension medium was added, and samples were gently pipetted on ice. After thorough dissociation, the suspension was filtered through a 70-μm nylon mesh, followed by centrifugation at 4°C for 10 minutes. Cells were loaded into the 10x Chromium system, aiming for a recovery of 10,000 cells. Library construction was carried out according to the Chromium Next GEM Single Cell 3’ Reagent Kits v3.1 (Dual Index) protocol provided by the manufacturer. Sequencing was performed using the NovaSeq X Plus platform.

### scRNA-seq analysis.

Raw read quality control was conducted using 10x Genomics Cell Ranger 7.0.1. Sample alignment to the reference genome GRCm38 (mm10), read quantification, and barcode filtering were performed with Cell Ranger Count. Data preprocessing and analysis were carried out in Seurat version 4.9.9. Cells were retained if they contained at least 500 detected genes and exhibited < 10% mitochondrial content. Normalization was conducted using the SCTransform method. PBS and CFA datasets were integrated via the FindIntegrationAnchors function. Principal component analysis (PCA) was performed, selecting the top 200 principal components for dimensionality reduction using the Uniform Manifold Approximation and Projection (UMAP) algorithm. Clustering was executed with the FindClusters function at a resolution of 1.5. Marker gene identification was performed by comparing each cluster against all others using the FindAllMarkers function, with a log-fold change threshold of 0.25 and inclusion criteria of > 25% cells expressing the gene.

## Figures and Tables

**Figure 5:** scRNA-Seq analysis reveals fibro-macrophage cell expansion in lymphatic deficient TMJ arthritis. (a) Schematic diagram of single-cell analysis. (b) UMAP visualization of major cell types highlighted with different colors in TMJ. Black arrowhead indicates increased macrophage population in mutant TMJ. (c) Proportion of each cell cluster in mutant group vs. control. (d) UMAP plot of macrophage-like immune cells reveals four subclusters, including M1 macrophages, Fibro-macrophages, M2 macrophages, and monocytes. (e) UMAP plot of macrophage-like immune cells for both control (red) and mutant (blue). (f) Volcano plot analysis of up- and down-regulated genes in fibro-macrophages compared to M1 macrophages. (g) Heatmap of signature genes in different macrophage cell clusters. (h) Feature plots showing the expression of *C1qa* (Macrophages), *Il-1β* (Inflammatory cytokines), and *Col1a1* (ECM), whose co-expression indicates the inflammatory and fibrosis-related fibro-macrophage expansion in lymphatic-deficient TMJ. (i) Brightfield imaging of sagittal sections stained with Masson’s Trichrome Staining in the superior regions of TMJ. Scale bar: 50 μm. (j, k) Quantification of the area fraction of collagen deposits in the superior area of TMJ.

## Data Availability

The scRNA-seq data generated in this study have been deposited in GEO under accession number GSE295404. Source data are provided with this paper.

## References

[1] Scrivani SJ, Keith DA, Kaban LB (2008) Temporomandibular disorders. N Engl J Med 359:2693–2705 19092154 10.1056/NEJMra0802472

[2] Valesan LF, Da-Cas CD, Réus JC, Denardin ACS, Garanhani RR, Bonotto D, Januzzi E, de Souza BDM (2021) Prevalence of temporomandibular joint disorders: a systematic review and meta-analysis. Clin Oral Invest 25:441–453 10.1007/s00784-020-03710-w 33409693

[3] Jariyasakulroj S, Shu Y, Lin Z, Chen J, Chang Q, Ko P-F, Chen J-F (2025) Mapping cell diversity and dynamics in inflammatory temporomandibular joint osteoarthritis with pain at single-cell resolution. JCI insight 10 10.1172/jci.insight.184379 PMC11948589 39927459

[4] Alstergren P, Pigg M, Kopp S (2018) Clinical diagnosis of temporomandibular joint arthritis. J Rehabil 45:269–281 10.1111/joor.12611 29392761

[5] International Classification of Orofacial Pain, 1st edition, ICOP (2020) (). Cephalalgia 40, 129–221. 10.1177/0333102419893823 32103673

[6] Ohrbach R, Bair E, Fillingim RB, Gonzalez Y, Gordon SM, Lim P-F, Ribeiro-Dasilva M, Diatchenko L, Dubner R, Greenspan JD (2013) Clinical orofacial characteristics associated with risk of first-onset TMD: the OPPERA prospective cohort study. J Pain 14:T33–T50 24275222 10.1016/j.jpain.2013.07.018 PMC3855658

[7] Ohrbach R, Dworkin S (2016) The evolution of TMD diagnosis: past, present, future. J Dent Res 95:1093–1101 27313164 10.1177/0022034516653922 PMC5004241

[8] Cooper BC, Kleinberg I (2007) Examination of a large patient population for the presence of symptoms and signs of temporomandibular disorders. CRANIO^®^ 25:114–126 17508632 10.1179/crn.2007.018

[9] Basbaum AI, Bautista DM, Scherrer G, Julius D (2009) Cellular and molecular mechanisms of pain. Cell 139:267–284 19837031 10.1016/j.cell.2009.09.028 PMC2852643

[10] Kuner R (2010) Central mechanisms of pathological pain. Nat Med 16:1258–1266 20948531 10.1038/nm.2231

[11] Kuner R, Flor H (2017) Structural plasticity and reorganisation in chronic pain. Nat Rev Neurosci 18:20–30 10.1038/nrn.2016.162 27974843

[12] Petrova TV, Koh GY (2020) Biological functions of lymphatic vessels. Science 369:eaax4063. 10.1126/science.aax4063 32646971

[13] Vaahtomeri K, Karaman S, Mäkinen T, Alitalo K (2017) Lymphangiogenesis guidance by paracrine and pericellular factors. Genes Dev 31:1615–1634 28947496 10.1101/gad.303776.117 PMC5647933

[14] Kim H, Kataru RP, Koh GY (2012) Regulation and implications of inflammatory lymphangiogenesis. Trends Immunol 33:350–356 22579522 10.1016/j.it.2012.03.006

[15] Skandalakis JE, Skandalakis LJ, Skandalakis PN (2007) Anatomy of the lymphatics. Surg Oncol Clin N Am 16:1–16 17336233 10.1016/j.soc.2006.10.006

[16] Escobedo N, Proulx ST, Karaman S, Dillard ME, Johnson N, Detmar M, Oliver G (2016) Restoration of lymphatic function rescues obesity in Prox1-haploinsufficient mice. JCI insight 1, e85096 26973883 10.1172/jci.insight.85096 PMC4786184

[17] Da Mesquita S, Louveau A, Vaccari A, Smirnov I, Cornelison RC, Kingsmore KM, Contarino C, Onengut-Gumuscu S, Farber E, Raper D (2018) Functional aspects of meningeal lymphatics in ageing and Alzheimer’s disease. Nature 560:185–191 30046111 10.1038/s41586-018-0368-8 PMC6085146

[18] Antila S, Karaman S, Nurmi H, Airavaara M, Voutilainen MH, Mathivet T, Chilov D, Li Z, Koppinen T, Park J-H (2017) Development and plasticity of meningeal lymphatic vessels. J Exp Med 214:3645–3667 29141865 10.1084/jem.20170391 PMC5716035

[19] Baluk P, Fuxe J, Hashizume H, Romano T, Lashnits E, Butz S, Vestweber D, Corada M, Molendini C, Dejana E (2007) Functionally specialized junctions between endothelial cells of lymphatic vessels. J Exp Med 204:2349–2362 17846148 10.1084/jem.20062596 PMC2118470

[20] Wiig H, Swartz MA (2012) Interstitial fluid and lymph formation and transport: physiological regulation and roles in inflammation and cancer. Physiol Rev 92:1005–1060 22811424 10.1152/physrev.00037.2011

[21] Breslin JW (2014) Mechanical forces and lymphatic transport. Microvasc Res 96:46–54 25107458 10.1016/j.mvr.2014.07.013 PMC4267889

[22] Petkova M, Kraft M, Stritt S, Martinez-Corral I, Ortsäter H, Vanlandewijck M, Jakic B, Baselga E, Castillo SD, Graupera M (2023) Immune-interacting lymphatic endothelial subtype at capillary terminals drives lymphatic malformation. J Exp Med 220:e20220741 36688917 10.1084/jem.20220741 PMC9884640

[23] Zhou S, Zhao G, Chen R, Li Y, Huang J, Kuang L, Zhang D, Li Z, Xu H, Xiang W (2024) Lymphatic vessels: roles and potential therapeutic intervention in rheumatoid arthritis and osteoarthritis. Theranostics 14:265 38164153 10.7150/thno.90940 PMC10750203

[24] Walsh D, Verghese P, Cook G, McWilliams D, Mapp P, Ashraf S, Wilson D (2012) Lymphatic vessels in osteoarthritic human knees. Osteoarthr Cartil 20:405–412 10.1016/j.joca.2012.01.012 22326896

[25] Bouta EM, Li J, Ju Y, Brown EB, Ritchlin CT, Xing L, Schwarz EM (2015) The role of the lymphatic system in inflammatory-erosive arthritis. (Elsevier), pp. 90–97 10.1016/j.semcdb.2015.01.001 PMC4397133 25598390

[26] Cao M, Ong M, Yung P, Tuan R, Jiang Y (2022) Role of synovial lymphatic function in osteoarthritis. Osteoarthr Cartil 30:1186–1197 10.1016/j.joca.2022.04.003 35487439

[27] Zhao Y, An Y, Zhou L, Wu F, Wu G, Wang J, Chen L (2022) Animal models of temporomandibular joint osteoarthritis: classification and selection. Front Physiol 13:859517 35574432 10.3389/fphys.2022.859517 PMC9095932

[28] Bordoni B, Varacallo M (2019) Anatomy, head and neck. temporomandibular joint

[29] Shi J, Liang Q, Zuscik M, Shen J, Chen D, Xu H, Wang YJ, Chen Y, Wood RW, Li J (2014) Distribution and alteration of lymphatic vessels in knee joints of normal and osteoarthritic mice. Arthritis Rheumatol 66:657–666 24574226 10.1002/art.38278 PMC4074307

[30] Cahill TJ, Sun X, Ravaud C, del Villa C, Klaourakis K, Lupu I-E, Lord AM, Browne C, Jacobsen SEW, Greaves DR (2021) Tissue-resident macrophages regulate lymphatic vessel growth and patterning in the developing heart. Development 148, dev194563 33462113 10.1242/dev.194563 PMC7875498

[31] Nygaard G, Firestein GS (2020) Restoring synovial homeostasis in rheumatoid arthritis by targeting fibroblast-like synoviocytes. Nat Rev Rheumatol 16:316–333 32393826 10.1038/s41584-020-0413-5 PMC7987137

[32] Eng C-HL, Lawson M, Zhu Q, Dries R, Koulena N, Takei Y, Yun J, Cronin C, Karp C, Yuan G-C (2019) Transcriptome-scale super-resolved imaging in tissues by RNA seqFISH+. Nature 568:235–239 30911168 10.1038/s41586-019-1049-y PMC6544023

[33] Xiang M, Grosso RA, Takeda A, Pan J, Bekkhus T, Brulois K, Dermadi D, Nordling S, Vanlandewijck M, Jalkanen S (2020) A single-cell transcriptional roadmap of the mouse and human lymph node lymphatic vasculature. Front Cardiovasc Med 7:52 32426372 10.3389/fcvm.2020.00052 PMC7204639

[34] Kim H, Kataru RP, Koh GY (2014) Inflammation-associated lymphangiogenesis: a double-edged sword? J Clin Investig 124:936–942 24590279 10.1172/JCI71607 PMC3938274

[35] Jariyasakulroj S, Chang Q, Ko P-F, Shu Y, Lin Z, Chen J, Chen J-F (2024) Temporomandibular joint pain measurement by bite force and Von Frey filament assays in mice. J visualized experiments: JoVE. 10.3791/67203 PMC11948272 39345152

[36] Karsdal MA, Nielsen SH, Leeming D, Langholm L, Nielsen M, Manon-Jensen T, Siebuhr A, Gudmann N, Rønnow S, Sand J (2017) The good and the bad collagens of fibrosis–their role in signaling and organ function. Adv Drug Deliv Rev 121:43–56 28736303 10.1016/j.addr.2017.07.014

[37] Ruscitto A, Chen P, Tosa I, Wang Z, Zhou G, Safina I, Wei R, Morel MM, Koch A, Forman M (2023) Lgr5-expressing secretory cells form a Wnt inhibitory niche in cartilage critical for chondrocyte identity. Cell stem cell 30, 1179–1198. e1177 37683603 10.1016/j.stem.2023.08.004 PMC10790417

[38] Alitalo K (2011) The lymphatic vasculature in disease. Nat Med 17:1371–1380 22064427 10.1038/nm.2545

[39] Joukov V, Kumar V, Sorsa T, Arighi E, Weich H, Saksela O, Alitalo K (1998) A recombinant mutant vascular endothelial growth factor-C that has lost vascular endothelial growth factor receptor-2 binding, activation, and vascular permeability activities. J Biol Chem 273:6599–6602 9506953 10.1074/jbc.273.12.6599

[40] Lin X, Xing L (2025) Using lightsheet microscopy to investigate the initial lymphatic network in the murine knee joints. bioRxiv, 2025.2003. 2021.644620

[41] Huang J, Liao C, Yang J, Zhang L (2024) The role of vascular and lymphatic networks in bone and joint homeostasis and pathology. Front Endocrinol 15:1465816 10.3389/fendo.2024.1465816 PMC11422228 39324127

[42] Buckley CD, Gilroy DW, Serhan CN, Stockinger B, Tak PP (2013) The resolution of inflammation. Nat Rev Immunol 13:59–66 23197111 10.1038/nri3362

[43] Serhan CN, Savill J (2005) Resolution of inflammation: the beginning programs the end. Nat Immunol 6:1191–1197 16369558 10.1038/ni1276

[44] Tuckey B, Srbely J, Rigney G, Vythilingam M, Shah J (2021) Impaired lymphatic drainage and interstitial inflammatory stasis in chronic musculoskeletal and idiopathic pain syndromes: exploring a novel mechanism. Front Pain Res 2:691740 10.3389/fpain.2021.691740 PMC8915610 35295453

[45] Remst DF, Blaney Davidson EN, van der Kraan PM (2015) Unravelling osteoarthritis-related synovial fibrosis: a step closer to solving joint stiffness. Rheumatology 54:1954–1963 26175472 10.1093/rheumatology/kev228

[46] Rim YA, Ju JH (2020) The role of fibrosis in osteoarthritis progression. Life 11:3 33374529 10.3390/life11010003 PMC7822172

[47] Brewer CM, Nelson BR, Wakenight P, Collins SJ, Okamura DM, Dong XR, Mahoney WM, McKenna A, Shendure J, Timms A (2021) Adaptations in Hippo-Yap signaling and myofibroblast fate underlie scar-free ear appendage wound healing in spiny mice. Developmental cell 56, 2722–2740. e2726 34610329 10.1016/j.devcel.2021.09.008 PMC8623355

[48] Pei G, Yao Y, Yang Q, Wang M, Wang Y, Wu J, Wang P, Li Y, Zhu F, Yang J (2019) Lymphangiogenesis in kidney and lymph node mediates renal inflammation and fibrosis. Science advances 5, eaaw5075 31249871 10.1126/sciadv.aaw5075 PMC6594767

[49] Feng S-Y, Lei J, Li Y-X, Shi W-G, Wang R-R, Yap AU, Wang Y-X, Fu K-Y (2022) Increased joint loading induces subchondral bone loss of the temporomandibular joint via the RANTES-CCRs-Akt2 axis. JCI insight 7:e158874 36173680 10.1172/jci.insight.158874 PMC9675482

